# Risk Pathways Contributing to the Alcohol Harm Paradox: Socioeconomic Deprivation Confers Susceptibility to Alcohol Dependence *via* Greater Exposure to Aversive Experience, Internalizing Symptoms and Drinking to Cope

**DOI:** 10.3389/fnbeh.2022.821693

**Published:** 2022-02-14

**Authors:** Ruichong Shuai, Justin J. Anker, Adrian J. Bravo, Matt G. Kushner, Lee Hogarth

**Affiliations:** ^1^School of Psychology, University of Exeter, Exeter, United Kingdom; ^2^Department of Psychiatry, University of Minnesota, Minneapolis, MN, United States; ^3^Department of Psychological Sciences, College of William & Mary, Williamsburg, VA, United States

**Keywords:** socioeconomic deprivation, aversive experience, mental health, coping motives, alcohol harm paradox

## Abstract

Socioeconomic deprivation is associated with greater alcohol problems despite lower alcohol consumption, but the mechanisms underpinning this alcohol harm paradox remain obscure. Fragmented published evidence collectively supports a multistage causal risk pathway wherein socioeconomic deprivation increases the probability of exposure to aversive experience, which promotes internalizing symptoms (depression and anxiety), which promotes drinking alcohol to cope with negative affect, which in turn accelerates the transition from alcohol use to dependence. To evaluate this proposed risk pathway, 219 hazardous drinkers from an undergraduate population completed questionnaires assessing these constructs in a single, cross sectional, online survey. Partial correlation coefficients revealed that each variable showed the strongest unique association with the next variable in the proposed multistage model, when adjusting for the other variables. Bootstrapped serial mediation analysis revealed that the indirect pathway linking all the variables in the proposed serial order was significant, while all other permutations were non-significant. Network centrality analysis corroborated the serial order of this indirect path. Finally, risk ratios estimated by categorizing the variables suggested that socioeconomic deprivation increased the risk of aversive experience by 32%, which increased the risk of internalizing symptoms by 180%, which increased the risk of drinking to cope by 64%, which increased susceptibility to alcohol dependence by 59%. These preliminary findings need to be corroborated by future research, nevertheless, they call for prevention strategies founded on social justice and the minimization of aversive experience in socially deprived individuals to mitigate mental health problems, maladaptive coping and addiction.

## Introduction

Nationally representative, cross-sectional and prospective surveys indicate that socioeconomically deprived individuals (i.e., people in lower socioeconomic statuses) drink less or the same amount of alcohol than less deprived individuals (Jefferis et al., 2007; Robinson and Harris, 2009; Fuller, 2013), but paradoxically, have greater alcohol related morbidity, mortality, injuries and alcohol dependence, even when controlling for alcohol consumption (Yang et al., 2007; Gauffin et al., 2013; Bellis et al., 2016; Møller et al., 2019). This *alcohol harm paradox* suggests that socioeconomically deprived individuals are more susceptible to transitioning from alcohol use to dependence due to risk factors other than average drinking level. There are at least 41 distinct theoretical explanations for why socioeconomically deprived individuals might be more susceptible to alcohol dependence (Boyd et al., 2021), including binge drinking pattern (Lewer et al., 2016), co-use of other substances (e.g., smoking and drinking), comorbid internalizing symptoms and drinking to cope (Kassel et al., 2000; Kushner et al., 2011; Merrill et al., 2014), all of which might promote dependence liability (Bellis et al., 2016).

This current article seeks to evaluate just one multistage sequential risk pathway that has been argued to contribute to the alcohol harm paradox. This pathway can be inferred from fragmented published evidence described in the following paragraphs. The studies below suggest that socioeconomic deprivation increases the probability of being exposed to aversive experiences (e.g., stress, trauma, bullying, discrimination, noise pollution, etc.), which increases the risk of internalizing symptoms (i.e., anxiety and depression), which establishes the conditions to learn that alcohol can be used to cope with (or dampen) negative affect, which confers susceptibility to transition more readily from alcohol use to dependence. This proposed sequence has not been tested in full by any single empirical method, but rather, must be inferred from a triangulation of methods, each with its own limitations as follows.

Human experimental studies have supported the proposed model by showing that motivation to drink alcohol, eat junk food and gamble can be increased by exposure to scarcity narratives which model socioeconomic deprivation (Callan et al., 2011; Snider et al., 2020), by stress induction procedures which model aversive environmental experience or anxiety (Shuai et al., 2020) and by sadness induction procedures which model depression symptoms (Hardy and Hogarth, 2017). Furthermore, these priming effects are amplified in individuals who drink to cope with negative affect (Hogarth and Field, 2020). These findings support the model by demonstrating that several of the predicted risk variables do cause an increase in substance motivation. However, laboratory paradigms have uncertain ecological validity and typically study only one risk variable at a time [although see the causal chain approach adopted by Callan et al. (2011)]. Therefore, epidemiological research is needed to capture “natural” variation in multiple risk and outcome variables simultaneously, to provide corroborating support for the multistage etiological processes underpinning the alcohol harm paradox.

Longitudinal studies have supported the proposed model by demonstrating that the risk and outcome variables occur in the predicted temporal order. Specifically, socioeconomic deprivation prospectively predicts aversive environmental experiences (Frioux et al., 2014; Imran et al., 2019), internalizing symptom severity (Ribeiro et al., 2017; Boyle, 2020), drinking to cope (Martin et al., 2019), and alcohol dependence (Gauffin et al., 2013). In turn, aversive experience (specifically, abuse, and bullying) predicts internalizing symptoms (Norman et al., 2012), drinking to cope (Topper et al., 2011), and substance dependence (Capusan et al., 2021). Internalizing symptom severity prospectively predicts drinking to cope (Windle and Windle, 2012; Young et al., 2015; Collins et al., 2018; Corcoran et al., 2021) and alcohol dependence (Anker and Kushner, 2019). Finally, drinking to cope prospectively predicts alcohol dependence (Menary et al., 2011; Crum et al., 2013). Thus, longitudinal studies are consistent with the chronological order of the variables in the proposed multistage model. However, no longitudinal design has captured the total model in a single analysis, perhaps because it would be necessary to know exactly when each variable appears – a challenging prospect if there are many variables and developmental progression differs between individuals.

Several longitudinal mediation studies have captured larger segments of the proposed model by demonstrating that the link between a predictor and outcome variable is explained by the expected intervening/mediating variable(s). Specifically, the prospective relationship between socioeconomic deprivation and substance use/dependence is mediated by exposure to less rewarding environments (Andrabi et al., 2017; Lee et al., 2018), internalizing symptoms (Segrin et al., 2021), and drinking to cope (Martin et al., 2019). The prospective relationship between an aversive experience [i.e., bullying (Topper et al., 2011) and partner violence (Øverup et al., 2015)] and alcohol dependence (controlling for consumption) is mediated by drinking to cope. The prospective relationship between internalizing symptoms [i.e., shyness (Young et al., 2015) and social anxiety (Collins et al., 2018)] and alcohol dependence (controlling for consumption) is mediated by drinking to cope. Finally, in a *serial* longitudinal mediation study, the prospective relationship between aversive experience (i.e., child sexual assault) and alcohol dependence was mediated by internalizing symptoms and drinking to cope in serial order (Hannan et al., 2017). Although supportive, none of these studies has captured the total model in a single design.

Cross-sectional designs are often dismissed as the weakest form of evidence in causal modeling. However, because the measurement timepoint occurs after all the relevant variables have manifested, although temporal order is lost, the potential to detect all of these variables in a single design is increased (Lowry and Gaskin, 2014; Jose, 2016; Fairchild and McDaniel, 2017; Pieters, 2017; Spector, 2019). At least 30 cross-sectional *single* mediation studies corroborate the model by demonstrating that substance use coping motives mediate the link that aversive experience (abuse, trauma) and internalizing symptoms share with substance dependence [see Table 1 of Hogarth (2020)]. Additionally, four cross-sectional *serial* mediation studies support the serial order of multiple mediating variables. Specifically, Peirce et al. (1994) found that the relationship between chronic financial strain and alcohol dependence was mediated by depression and drinking to cope, in turn. Likewise, Desalu et al. (2019) found that among black college students, the relationship between racial discrimination (an aversive experience) and alcohol problems was serially mediated by depression symptoms and drinking to cope, in turn. Similarly, Park et al. (2019) found that the relationship between childhood maltreatment and alcohol problems was serially mediated by anxiety symptoms and drinking to cope, in turn. Finally, McPhee et al. (2020) found that the relationship between reduced rewarding experience during COVID-19 lockdown and increased alcohol consumption was serially mediated by depression symptoms and drinking to cope, in turn.

Although the foregoing studies collectively support the proposed multistage causal model underlying the alcohol harm paradox, no study has measured all the relevant variables simultaneously to test whether the unique associations fit the model. The current study aimed to measure all the variables simultaneously in a single cross-sectional survey completed by young adult hazardous drinkers. It was expected that in partial correlations, each variable would show the strongest unique association with the next variable in the proposed chain controlling for all other variables. Furthermore, serial mediation analysis was expected to reveal that each variable incrementally predicted the next variable in the proposed chain when controlling for prior variables, consistent with the proposed serial order. Moreover, examination of the indirect pathways (the product of these incremental associations) was expected to reveal that socioeconomic deprivation was associated with susceptibility to alcohol dependence through aversive experience, internalizing symptoms and drinking to cope in turn, but not through other permutations of these mediators. Finally, centrality closeness scores derived from network analysis were expected to confirm that socioeconomic deprivation was connected to alcohol susceptibility through these three mediators, with internalizing symptoms as the most central node. These findings would converge with the fragmented longitudinal and experimental evidence cited earlier, to support a holistic multistage causal account of the alcohol harm paradox.

## Materials and Methods

### Participants

Participants were recruited for different research projects from the Psychology research pool at Exeter, the Facebook page Overheard at Exeter, and Exeter 10,000/Peninsula Research Bank. A total of 512 participants who completed the same set of measures were compiled together. The analytical sample for the present study compromised of 219 aged 18–25 who reported past year hazardous drinking [hazardous drinking was defined by total score of ≥8 for males and ≥7 for females on the Alcohol Use Disorders Identification Test (Babor et al., 2001)]. The restriction of analytical sample ensured that the theoretical model could be applied to hazardous drinkers and to make AUDIT scores linear (by excluding the cluster of mild and zero drinkers), so the data met the homoscedasticity requirement for multiple regression (see section “Analytical Plan”). The analyzed sample had a mean age of 19.84 (SD = 1.32) and comprised of majority females (83.6%). Table 1 shows the mean scores for scales selected for the analysis. Participants provided informed consent, were debriefed and reimbursed with course credits or a £3 Amazon voucher depending on their wishes. The study was approved by the School of Psychology Research Ethics Committee.

**TABLE 1:** Correlations and sample characteristics.

Bivariate Pearson correlations					
Methods	1	2	3	4	Mean (SD, range)
1. Socioeconomic deprivation	–				3.50 (1.04, 2–5)
2. Aversive experience	0.22**	–			2.28 (0.53, 1–3.44)
3. Internalizing symptoms	0.10	0.65**	–		8.19 (5.26, 0–22.50)
4. Drinking to cope	0.11	0.34**	0.39**	–	3.53 (2.12, 0–9.44)
5. Alcohol susceptibility	−0.001	0.21**	0.28**	0.33**	0.49 (1.16, −2.08 to 3.50)

**Partial correlations**					
**Methods**	**1**	**2**	**3**	**4**	

1. Socioeconomic deprivation	–				
2. Aversive experience	0.11	–			
3. Internalizing symptoms	–	0.50	–		
4. Drinking to cope	–	0.09	0.17	–	
5. Alcohol susceptibility	–	–	0.09	0.18	

Bivariate Pearson correlation coefficients (top) and partial correlation coefficients (bottom) for key variables in the proposed sequential model. The right hand column shows the mean questionnaire scores of the sample. The questionnaires used to measure each construct are listed in the “Materials and Methods” section. The Bivariate Pearson correlation coefficients in the top half of the table quantify the relationships between variables in the model: significant correlations are emboldened, **p < 0.01. The mean, SD and range of variables are shown in the right-hand column. The partial correlation coefficients in the bottom half of the table quantify unique relationships controlling for all other variables. As predicted, each variable showed the strongest unique association (partial correlation) with the next variables in the proposed model. That is, socioeconomic deprivation was only associated with aversive experience, which was most strongly associated with internalizing symptoms, which was most strongly associated with drinking to cope, which was most strongly associated with susceptibility to transition from alcohol use to dependence.

### Questionnaires

Questionnaires were assessed in a single, cross sectional, online survey in the following order. Socioeconomic deprivation was measured with the MacArthur Scale of Subjective Social Status adapted from Goodman et al. (2001), which is a single item stating “Imagine the scale below represents how society is set up. On the left are the people who are best off in terms of money, schooling, jobs and respect. On the right are the people who are worst off. Please tell us where you think the family you grew up in falls on this scale.” Responses ranged from 1 “best off family” to 9 “worst off family,” so higher scores notionally capture early experience subjective relative familial poverty (coined socioeconomic deprivation for the purposes of this study), which occurred before the other variables in the model (although retrospective reports can be influenced by recent experience). The validity of this scale for the current purposes is supported by its’ association with household income, substance use and internalizing symptoms (Finch et al., 2013; Hawley, 2020).

Aversive experience was assessed with the Reward Probability Index (RPI; Carvalho et al., 2011), which comprises of 20 items divided into two subscales. The 9-item Environmental Suppressors subscale (hereafter “aversive experience”) measures aversive experiences (α = 0.82), with the strongest loaded items in the cited factor analysis being “I have had many unpleasant experiences” and “It seems like bad things always happen to me.” The Reward Probability subscale (hereafter “reward probability”) measures the ability to obtain/experience reward (α = 0.82), with the strongest loaded items being “I have the abilities to obtain pleasure in life” and “I feel a strong sense of achievement.” Participants endorsed each item on a scale ranging from 1 “*Strongly disagree*” to 4 “*Strongly agree*.” The validity of the Environmental Suppressors subscale measure of aversive experience for the current analytical purposes is supported by its unique association with adverse childhood experiences and with income and alcohol dependence (controlling for consumption) over the Reward Probability subscale (Loomis, 2020; Voss et al., 2021).

Internalizing symptoms were measured with the Patient Health Questionnaire Depression Scale (PHQ-8; Kroenke et al., 2009) and the Generalized Anxiety Disorder Questionnaire (GAD-7; Löwe et al., 2008). The PHQ-8 contains eight items (e.g., “little interest or pleasures in doing things”), and the GAD-7 contains 7 items (e.g., “feeling nervous, anxious or on edge”) which participants endorsed on a scale from 0 “*Not at all*” to 3 “*Nearly every day*.” The two scale mean scores were strongly correlated (*r* = 0.75, *p* < 0.001), so they were averaged to create a single score for internalizing symptoms (α = 0.94). A score of 10 marks the boundary between mild and moderate symptom severity.

The Drinking Motives were measured with the Modified Drinking Questionnaire-Revised [DMQR validated by Grant et al. (2007)], which contains five subscales assessing drinking to cope with anxiety (e.g., “to relax”), and depression (e.g., “to numb my pain”), to drink for pleasure enhancement (e.g., “to get a high,” α = 0.82), for conformity (e.g., “to be liked,” α = 0.85), and to be social (e.g., “as a way to celebrate,” α = 0.77), which participants endorsed on a scale ranging from 0 “*never*” to 10 “*always*.” The coping with anxiety/depression subscales were highly correlated (*r* = 0.70, *p* < 0.001), so they were averaged to create a single drinking to cope score (α = 0.94) as done in prior research (Bravo and Pearson, 2017).

Alcohol use and dependence were measured with the 10-item Alcohol Use Disorder Identification Test (AUDIT) assessing past 12-month experience (Babor et al., 2001). As noted earlier, total scores range from 0 to 40, with hazardous drinking defined ≥8 for males and ≥7 for females. Validation studies indicate that there are two subscales of the AUDIT (Maisto et al., 2000; Doyle et al., 2007). The Consumption subscale (α = 0.42) is assessed by three items: “How often do you have a drink containing alcohol,” “How many standard drinks do you have on a typical day when you are drinking,” “How often do you have six or more standard drinks on one occasion.” The Consequences subscale (α = 0.41) is assessed by seven items addressing the loss of control over drinking (e.g., “How often during the last year have you found that you were not able to stop drinking once you had started”) and alcohol-related problems (e.g., “How often during the last year have you failed to do what was normally expected from you because of drinking”). The dependent variable in the present analyses was the *alcohol susceptibility score* – the standardized residual of the consequences subscale over the consumption subscale. Higher scores indicate that participants have more alcohol consequences than would be predicted based on their level of consumption, estimating their susceptibility to transition from alcohol use to dependence. These scores are akin to z scores in having a mean zero with 68% of the sample falling between +1 and −1 and 95% within +2 and −2.

### Analytical Plan

IBM SPSS Statistics 28 and JASP software were used for data analyses. Assumption checks and outlier correction procedures were as follows. Alcohol susceptibility scores were found to meet the homoscedasticity assumption for multiple regression in relation to the predictor variables listed in Table 1, using the Breusch–Pagan and Konker Test, *X*^2^ (1, 217) = 2.38, *p* = 0.123. Mahalanobis distance indicated that there were no multivariate outliers. Univariate outliers on any of the variables (>1.5 times the interquartile range) were winsorized to match the nearest non-outlying score (four data points for socioeconomic deprivation and two data points for alcohol susceptibility were corrected), ensuring that outliers did not influence regression models.

A Pearson bivariate correlation matrix tested the unadjusted relationships between the main variables: socioeconomic deprivation, aversive experience, internalizing symptoms, drinking to cope and alcohol susceptibility (Table 1 top). A partial correlation matrix then tested the unique relationships between variables, adjusting for all other variables (Table 1 bottom). It was predicted that each variable would show the strongest unique association with the next variable in the proposed multistage model. The partial coefficients were then shrunk (sparsified) using Extended Bayesian Information Criterion Graph Least Absolute Shrinkage and Selection Operator (EBICglasso), such that small (spurious) coefficients were shrunken to be exactly zero and omitted from the matrix table. The remaining partial coefficient values which are greater than zero can be deemed significant. These partial coefficient were then used to conduct network centrality analysis using JASP software to calculate the “closeness” centrality score for each variable (node) in the model. Closeness scores quantify the sum of all shortest paths (number of hops) from the variable (node) of interest to every other variable in the network (following the non-zero partial coefficients or edges). JASP produces the inverse of closeness scores so that a higher closeness centrality score indicates that this node is more central to the network. These closeness values provide another approach to corroborate the serial order of variables in the proposed etiological model.

Then, serial mediation analysis was conducted using PROCESS software for SPSS (v.3.5 model 6), with socioeconomic deprivation as the predictor variable X, aversive experience, internalizing symptoms and drinking to cope as the three serial mediators M1, M2, and M3, and alcohol susceptibility as the outcome variable Y. Beta values in this model quantify the incremental prediction of the following variable in the chain controlling for prior variables. We examined the total, direct, and indirect effects of each predictor using percentile-based bootstrapped estimates based on 10,000 bootstrapped samples. Statistical significance was determined by 95% percentile-based bootstrapped confidence intervals that do not contain zero. It was expected that only the predicted indirect path from socioeconomic deprivation to alcohol susceptibility through all three mediators would be significant, supporting the proposed model. Finally, the same model was rerun adjusting for six covariates separately (age, gender, the Reward Probability subscale of the RPI and the other DMQR drinking motives) to test whether the predicted indirect pathway remained significant when controlling for the effect of these covariates on the outcome variable.

Finally, risk ratios (with confidence intervals) were calculated to estimate the risk (probability) that an individual would be exposed to the next risk variable if exposed to the previous risk variable in the proposed causal chain. Specifically, participants were categorized into higher and lower subgroups around the median for each of the first four variables – socioeconomic deprivation, aversive experience, internalizing symptoms, drinking to cope – and categorized as being susceptibility to alcohol dependence if their alcohol susceptibility score was >0, or not susceptible if their score was ≤0. Risk ratios represented the proportionate risk of being categorized in the higher subgroup for each variable depending on the membership of the subgroups of the previous variable in the chain.

## Results

### Correlation and Partial Correlation Matrices

Table 1 shows descriptive statistics (top), the bivariate Pearson correlation matrix (top), and the partial correlation matrix (bottom) between the variables in the proposed model. The results corroborated hypotheses such that each variable showed the strongest association (correlations) and unique association controlling for other variables (partial correlations) with the next variable in the proposed model, revealing the serial pathway of associations linking socioeconomic deprivation – aversive experience – internalizing symptoms – drinking to cope – alcohol susceptibility. This pathway supports the proposed multistage account of the alcohol harm paradox.

### Serial Mediation Analysis

**FIGURE 1:**
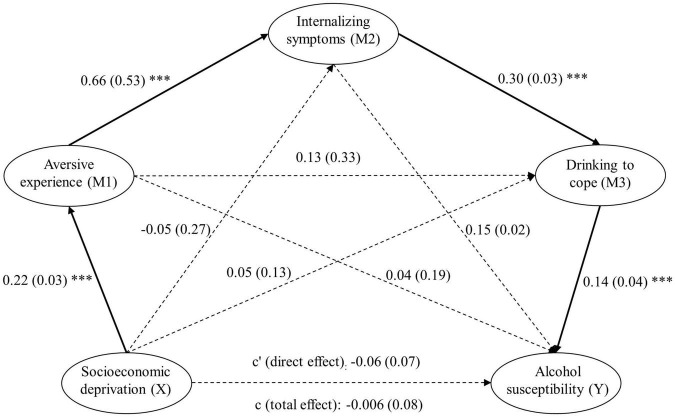
Serial mediation model testing the proposed multistage account of the alcohol harm paradox (*N* = 219). For each connecting line, the standardized beta value (i.e., the strength of the unique association) between the two variables is shown (controlling for any proceeding variables in the chain). The bootstrapped standard error of each beta value is shown in brackets. Significant beta values are emphasized by complete connecting lines and labeled as ***p < 0.001. Dashed lines connect non-significant beta values. As predicted, each variable showed a significant incremental association with the next variable in the chain linking socioeconomic deprivation to alcohol susceptibility. Furthermore, the only significant indirect pathway (product of the beta values) connected socioeconomic deprivation to alcohol susceptibility was through this specific pathway and no other (see Table 2).

Figure 1 shows the serial mediation model. The relationship between variables is represented by standardized beta values, which quantify the strength of the unique relationship between variables, controlling for (i.e., over and above) any proceeding variable in the chain. The total effect (C path) of socioeconomic deprivation and alcohol susceptibility was not significant, somewhat contradicting the harm paradox. However, as predicted by the multistage model, there was a significant indirect path linking socioeconomic deprivation to alcohol susceptibility through aversive experience, internalizing symptoms and drinking to cope, in turn, shown at the bottom of Table 2. By contrast, there was no other significant indirect path linking socioeconomic to alcohol susceptibility via any other permutation of the mediators, supporting the specificity of the predicted indirect sequential pathway. Importantly, the significant predicted indirect pathway remained significant when the same serial mediation model was run separately for six covariates: age (*b* = 0.010, SE = 0.006, CI = 0.002–0.024), gender (*b* = 0.011, SE = 0.006, CI = 0.003–0.024), the Reward Probability index of the RPI (*b* = 0.005, SE = 0.004, CI = 0–0.014), DMQR social (*b* = 0.008, SE = 0.005, CI = 0.0006–0.020), DMQR enhancement (*b* = 0.013, SE = 0.006, CI = 0.004–0.028), and DMQR conformity (*b* = 0.006, SE = 0.004, CI = 0.0002–0.016). These findings indicate that the predicted indirect path remained significant when the effect of these covariates on the outcome measure was controlled for, suggesting the path could not be explained by these covariates, i.e., the path was specific to the selected variables.

**TABLE 2:** Summary of indirect pathway tested in key variables.

Indirect effects	Standardized coefficients	Standard error	Confidence interval
x - > m1 - > y	0.008	0.021	−0.032 to 0.051
x - > m2 - > y	−0.008	0.011	−0.034 to 0.010
x - > m3 - > y	0.014	0.019	−0.022 to 0.054
x - > m1 - > m2 - > y	0.023	0.016	−0.002 to 0.059
x - > m1 - > m3 - > y	0.008	0.006	−0.002 to 0.023
x - > m2 - > m3 - > y	−0.004	0.005	−0.017 to 0.005
**x - > m1 - > m2 - > m3 - > y**	**0.012**	**0.006**	**0.003 to 0.026**

*Indirect pathways were tested between socioeconomic deprivation (x) and alcohol susceptibility (y) through aversive experience (m1), internalizing symptoms (m2), and drinking to cope (m3). The only significant indirect serial pathway is emboldened, linking socioeconomic deprivation to alcohol susceptibility via aversive experience, internalizing symptom severity, and drinking to cope, in turn. All other indirect pathways were non-significant.*

### Network Analysis

**FIGURE 2:**
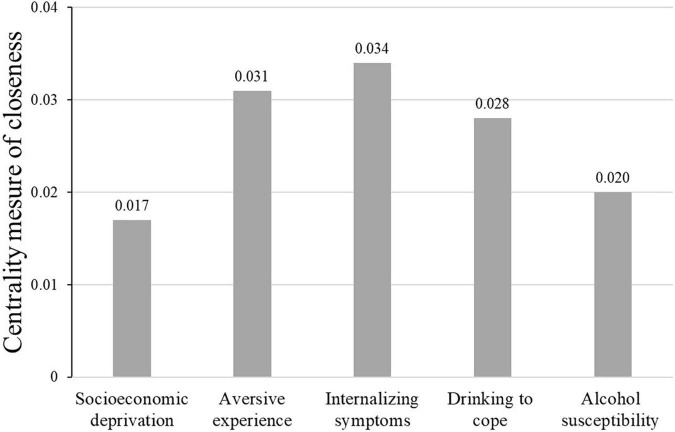
Centrality measure of closeness derived from network analysis. The values reported in the figure are the inverse of the sum of all shortest paths from the variable (node) of interest to every other variable in the network, so that higher centrality closeness scores indicate more centrality in the model. As predicted by the model, socioeconomic deprivation and susceptibility to alcohol dependence were outlying in the network and connected via the three mediators, with internalizing symptoms at the center.

Figure 2 shows the closeness centrality scores derived from network analysis, where higher scores indicate a shorter number of hops required for the variable to connect with all other variables in the network, following the non-zero partial coefficients in Table 1. These scores confirmed that socioeconomic deprivation and alcohol susceptibility were the most peripheral variables at either end of the network. By contrast, internalizing symptoms were the most central variable, while aversive experience and drinking to cope were slightly less central. These closeness scores are consistent with the proposed serial order of the variables in the multistage account of the alcohol harm paradox.

### Risk Ratios

**FIGURE 3:**
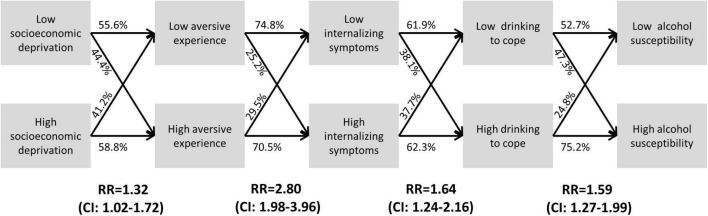
Estimating the individual risk of transitioning down the proposed pathway. Lines show the proportion of participants in the high/low group of each variable who fell within the high/low group of the next variables in the proposed model. For example, 58.8% of the high socioeconomic deprivation group versus 44.4% of the low socioeconomic deprivation group fell into the high aversive experience group. Risk ratios (RR) were calculated by dividing the proportion of the high versus low group who fell into the high group of the next variable (e.g., 58.8%/44.4% = 1.32). None of the confidence intervals contained 1 indicating that the risk ratios were all significant. The risk ratios suggest that high socioeconomic deprivation conferred a 32% increased risk of high aversive experience, which conferred an 180% increased risk of internalizing symptoms, which conferred a 64% increased risk of drinking to cope, which conferred a 59% increased susceptibility to alcohol dependence.

Figure 3 shows the risk ratios (with confidence intervals) of being exposed to each variable given exposure to the previous variable in the pathway. The numbers on the lines are the proportion of participants within the high and low subgroup of each variable in the low and high subgroup of the next variable in the chain. These values were used to calculate the risk ratios shown under each transition point. These risk ratios suggest that socioeconomic deprivation increased the risk of being in the higher aversive experience subgroup by 1.32 (32%, CI = 1.02–1.72), which increased the risk of being in the higher internalizing symptoms subgroup by 2.80 (180%, CI = 1.98–3.96), which increased the risk of being in the higher drinking to cope subgroup by 1.64 (64%, CI = 1.24–2.16), which increased the risk of being in the higher alcohol susceptibility subgroup by 1.59 (59%, CI = 1.27–1.99).

## Discussion

The current cross-sectional analyses of 219 hazardous, 18- to 25-year-old drinkers supported the predictions of the proposed multistage causal model underpinning the alcohol harm paradox. First, partial correlations confirmed that each variable showed the strongest unique association with the next variable in the proposed sequence. That is, socioeconomic deprivation was only associated with aversive experience, which was most strongly associated with internalizing symptoms, which was most strongly associated with drinking to cope, which was most strongly associated with susceptibility to transition from alcohol use to dependence (greater alcohol consequences adjusting for consumption). Second, serial mediation analysis further supported the model by showing that the indirect pathway (i.e., the product of the partial associations) linking the variables was significant only in the proposed sequential pathway, while all other indirect pathways through these variables were non-significant. Third, closeness centrality scores derived from network analysis supported the model by showing that socioeconomic deprivation and susceptibility to alcohol dependence were outlying in the network, and connected via the three mediators, with internalizing symptoms at the center, as predicted. Finally, risk ratios estimated by categorizing variables suggested that socioeconomic deprivation increased the risk of aversive experience by 32%, which increased the risk of internalizing symptoms by 180%, which increased the risk of drinking to cope by 64%, which increased susceptibility to alcohol dependence by 59%. These risk ratios provide heuristic effect size values for understanding the degree of risk an individual is exposed to of progressing through the causal chain culminating in alcohol dependence given exposure to any of the proceeding risk variables. Although the current data are preliminary and have limitations (see below), the multistage model described provides support for intervention strategies that emphasize improvements in social justice and the minimization of aversive experience to tackle mental health and alcohol problems.

The current cross-sectional findings corroborate, unify and extend the fragmented published evidence for the proposed model outlined in the Introduction. As noted, experimental evidence has demonstrated the causal effect of single risk variables on addictive behavior but provided little insight into how these variables link to form a natural etiological pathway. Longitudinal studies have confirmed the temporal order of the risk and outcome variables but have not incorporated them all into a single analysis, requiring between-experiment inferences to appreciate the total model. Similarly, mediation analyses (both longitudinal and cross-sectional) have demonstrated that intervening variables explained the link between risk and outcome variables as predicted by the model, but again, none of these analyses have encompassed all the variables simultaneously. The main contribution of the current analysis is to demonstrate in a single analytical sample that all the variables are indeed uniquely associated in the way anticipated by the model. However, because cross-sectional analyses provide the weakest support for causal inferences, the triangulation of all the evidence compiled here provides the best support for the proposed multistage causal model.

There are several limitations of the current analysis. The conclusions are based mainly on the responses of young university females, which limits the generalizability to other samples. Indeed, our sample compromised 83.6% females, and included only 36 males, meaning that gender differences in the risk pathways could not be meaningfully addressed. A priority for future research should be to evaluate the commonality of the risk pathways between genders in a sample sufficiently powered to test this question. Another important issue is the unknown chronology of the variables measured in the study. We have offered a developmental model based on unique associations from our cross-sectional analysis triangulated with published longitudinal and experimental data, which provides more information on temporal order and causal effects but has tested only fragmented portions of the model. The obvious next research step to strengthen the model would be to conduct a longitudinal design or find an existing cohort study with open access data or conduct retrospective life course analysis assessing all the relevant variables. This would establish the model’s reliability and provide more information on the timeframe (sensitive periods) when exposure to each variable confers the risk of experiencing the next variable in the chain.

There are two issues concerning the sampling and measurement of socioeconomic deprivation. First, there was no direct association between socioeconomic deprivation and susceptibility to alcohol dependence, in contrast to nationally representative data (Yang et al., 2007; Gauffin et al., 2013; Bellis et al., 2016). This could be due to the under-sampling of deprived individuals due to the predominantly sampled students being relatively wealthy (Chowdry et al., 2013), thus underestimating the association between socioeconomic deprivation and other variables. However, the absence of a direct association between socioeconomic deprivation and susceptibility to alcohol dependence does not negate the potential of mediation analysis to reveal the indirect pathway linking these variables (Hayes, 2009; Zhao et al., 2010; Rucker et al., 2011). The second issue is the single item used to measure socioeconomic deprivation, which reflected only participants’ relative childhood family status. Socioeconomic status comprises a wide diversity of dimensions including education, income (personal and familial), occupation, neighborhood, environment, experienced at different time points (i.e., childhood versus adult), which have been variously linked to alcohol dependence (Yang et al., 2007; Huckle et al., 2010; Karriker-Jaffe, 2011; Calling et al., 2019). Future work needs to address these two concerns by randomly sampling participants across the socioeconomic spectrum and comprehensively assessing socioeconomic deprivation to provide a more accurate and detailed account of its role within the risk pathway.

The third limitation is that the aversive experience measure (the environmental suppressors ES subscale of the RPI) could conflate environmental and internal experience, thus tapping essentially the same construct as the internalizing symptom measures. Some of the aversive experience items have face validity in specifically addressing external events (e.g., “It seems like bad things always happen to me”), while others are ambiguous (e.g., “I have had many unpleasant experiences”). However, there is some evidence that the aversive experience measure does uniquely tap external experience. In the current study, aversive experience correlated with socioeconomic deprivation whereas internalizing symptoms did not (see Table 1), suggesting unique sensitivity to external experience. Another study found that the aversive experience measure correlated with the number of adverse childhood experiences (ACEs) retrospectively reported prior to the age of 18 by university students (Loomis, 2020). The ACE items included familial abuse, neglect, poverty, divorce, mental illness and imprisonment. Similarly, unpublished data from our lab found that the aversive experience measure was correlated with the experience of being bullied. These findings validate the aversive experience measure as an index of aversive environmental experience. Indeed, one potentially valuable feature of the aversive experience measure is that it asks general questions about unpleasant experiences. By contrast, some authors have argued that other trauma questionnaires might underestimate aversive experience by listing specific traumatic events that might not match participants’ experience (Turner and Avison, 2003). The implication is that future studies should include alongside the aversive experience measure, a comprehensive assay of trauma exposure, to provide a more valid, accurate and detailed account of the role of this variable within the risk pathway.

The final issue concerns the alcohol susceptibility measure – the residual of alcohol consequences over the alcohol consumption subscales of the AUDIT. Although this measure is the same as some studies addressing the alcohol harm paradox (Beard et al., 2016), others have used more extensive, independent assays of alcohol consumption and consequences (Yang et al., 2007; Gauffin et al., 2013; Bellis et al., 2016; Møller et al., 2019). Separately, “telescoping” studies have taken an important further step in using the time between age of alcohol onset and the age of meeting dependence criteria (reported retrospectively) providing a more direct (face valid) assay of susceptibility to transition from use to dependence (Kushner et al., 2012; Oberleitner et al., 2015; Menary et al., 2017). Another issue is that the AUDIT subscales had low Cronbach’s alpha, suggesting poor correspondence between the items. The utility of Cronbach’s alpha in evaluating symptom checklists has been questioned (Sijtsma, 2009; Cho and Kim, 2015), because symptoms are additive and individuals may receive a diagnoses despite endorsing different symptom combinations (Lane and Sher, 2015). The poorer reliability of the AUDIT subscales in the current analysis compared to published validation studies (Selin, 2003; Shields and Caruso, 2003; Erford et al., 2021) might be due to the restriction of the sample to hazardous drinkers limiting the range of AUDIT scores (Fife et al., 2012). Consistent with this claim, the reliability of the consumption (α = 0.64) and consequences (α = 0.68) subscales was acceptable when the full sample (*N* = 512) was examined. Therefore, the recommendation for future work is to measure alcohol use and consequences more comprehensively in independent questionnaires and measure the time between the age of alcohol onset and dependence as a second corroborating index, to better support the argument that the risk pathway underpins susceptibility to transition from alcohol use to dependence.

To conclude, the study corroborated fragmented longitudinal and experimental studies by providing preliminary evidence that a multistage risk pathway may contribute to the alcohol harm paradox. This multistage risk pathway suggests that socioeconomic deprivation confers risk of alcohol dependence by increasing exposure to aversive experience, and thence to internalizing symptoms and drinking to cope, in turn. The model emphasizes the under-exploited opportunity for prevention strategies founded on social justice and the minimization of aversive experience in socially deprived individuals to mitigate mental health problems, maladaptive coping and addiction. Specifically, the model strengthens the growing challenge of the dominant biomedical approach to treating mental health (Kinderman, 2019; Boyle, 2020) and addiction (Alexander, 2008; Heyman, 2009; Hart, 2013; Hall et al., 2015; Hogarth, 2022). The core argument of these authors is that mental health and addiction are better understood as being driven by psychosocial factors, i.e., stressors and adversity linked to socioeconomic deprivation, rather than being driven by intrapersonal factors such as brain chemistry/structure or temperament. Crucially, these authors argue, biomedical interventions have failed to produce cost-effective solutions, and have misappropriated resources from structural reforms, which historically have produced better outcomes, for example, interventions that address health, nutrition, education, employment, wealth, housing, environment, etc. These authors aim to build an empirical case that provokes a transition in research and political focus toward structural reforms intentionally implemented to prevent mental health problems and addiction, as envisaged by the Health in All Policies agenda implemented by local government (Marmot and Seccombe, 2016). The present article supports this case by emphasizing the importance of socioeconomic deprivation and attendant aversive experience in the genesis of mental health problems, maladaptive coping and addiction. Although structural reforms are ambitious, they may prove to have greater long-term efficacy compared to perpetual, marginally effective remedial strategies to treat cases of addiction once they have arisen.

## Data Availability Statement

The raw data supporting the conclusions of this article will be made available by the authors, without undue reservation.

## Ethics Statement

The studies involving human participants were reviewed and approved by the School of Psychology Research Ethics Committee, University of Exeter. The participants provided their written informed consent to participate in this study.

## Author Contributions

RS contributed to conceptualization, methodology, investigation, data curation, formal analysis, and writing up original draft. JA, AB, and MK contributed to reviewing and editing. LH contributed to conceptualization, methodology, data curation, formal analysis, reviewing and editing, and supervision. All authors contributed to the article and approved the submitted version.

## Conflict of Interest

The authors declare that the research was conducted in the absence of any commercial or financial relationships that could be construed as a potential conflict of interest.

## Publisher’s Note

All claims expressed in this article are solely those of the authors and do not necessarily represent those of their affiliated organizations, or those of the publisher, the editors and the reviewers. Any product that may be evaluated in this article, or claim that may be made by its manufacturer, is not guaranteed or endorsed by the publisher.
